# Fungicide-Tolerant Plant Growth-Promoting Rhizobacteria Mitigate Physiological Disruption of White Radish Caused by Fungicides Used in the Field Cultivation

**DOI:** 10.3390/ijerph17197251

**Published:** 2020-10-04

**Authors:** Sadaf Khan, Mohammad Shahid, Mohammad Saghir Khan, Asad Syed, Ali H. Bahkali, Abdallah M. Elgorban, John Pichtel

**Affiliations:** 1 Department of Agricultural Microbiology, Faculty of Agricultural Sciences, Aligarh Muslim University, Aligarh 202002, India; sadaf4393@gmail.com (S.K.); khanms17@rediffmail.com (M.S.K.); 2 Department of Botany and Microbiology, College of Science, King Saud University, P.O. 2455, Riyadh 11451, Saudi Arabia; asadsayyed@gmail.com (A.S.); abahkali@ksu.edu.sa (A.H.B.); aelgorban@ksu.edu.sa (A.M.E.); 3 Natural Resources and Environmental Management, Ball State University, Muncie, IN 47306, USA; jpichtel@bsu.edu

**Keywords:** fungicide toxicity, *Raphanus sativus*, *Pseudomonas* spp., remediation, protein content

## Abstract

Excessive use of fungicides in agriculture may result in substantial accumulation of active residues in soil, which affect crop health and yield. We investigated the response of *Raphanus sativus* (white radish) to fungicides in soil and potential beneficial interactions of radish plants with fungicide-tolerant plant growth-promoting rhizobacteria (PGPR). The PGPR were isolated from cabbage and mustard rhizospheres. Morphological and biochemical characteristics measured using standard methods, together with analysis of partial 16S rRNA gene sequences, revealed that fungicide-tolerant PGPR, isolates PS3 and AZ2, were closely related to *Pseudomonas* spp. These PGPR survived in the presence of high fungicide concentrations i.e., up to 2400 μg mL^−1^ carbendazim (CBZM) and 3200 μg mL^−1^ hexaconazole (HEXA). Bacterial isolates produced plant growth stimulants even under fungicide stress, though fungicides induced surface morphological distortion and alteration in membrane permeability of these bacteria, which was proved by a set of microscopic observations. Fungicides considerably affected the germination efficiency, growth, and physiological development of *R. sativus*, but these effects were relieved when inoculated with PGPR isolates. For instance, CBZM at 1500 mg kg^−1^ decreased whole dry biomass by 71%, whole plant length by 54%, total chlorophyll by 50%, protein content by 61%, and carotenoid production by 29%. After applying isolate AZ2 for white radish grown in CBZM (10 mg kg^−1^)-amended soil, it could improve plant growth and development with increased whole plant dry weight (10%), entire plant length (13%) and total chlorophyll content (18%). Similarly, isolate PS3 enhanced plant survival by relieving plant stress with declined biomarkers, i.e., proline (12%), malondialdehyde (3%), ascorbate peroxidase (6.5%), catalase (18%), and glutathione reductase (4%). Application of isolates AZ2 and PS3 could be effective for remediation of fungicide-contaminated soil and for improving the cultivation of radish plants while minimizing inputs of fungicides.

## 1. Introduction

*Raphanus sativus* (white radish), a member of the Brassicaceae family, is an important vegetable crop in India and Southeast Asia; both leaves and tubers of the radish plant are consumed. It is a rich source of vitamin A and vitamin C [1] and minerals like sulfur [2]. It is also used as a medicine to cure liver disorders and jaundice [3]. *R. sativus* is sensitive to be invaded by diverse phytopathogenic fungi and protists, causing several diseases, such as alternaria leaf blight, clubroot, downy mildew, fusarium wilt, and damping off, leading to extensive yield losses [4]. Also, a variety of soil-borne fungal pathogens were found responsible for reducing the yield of radish. Experiments performed in India to assess the yield loss of radish crop due to downy mildew caused by *Peronospora parasitica* showed that the maximum disease severity was at 48.6%, and resulting in 30.3% leaf defoliation with up to 39.6% seed loss [5].

In the modern agricultural era, fungicides are routinely applied in vegetable cropping systems to regulate the soil-borne plant diseases caused by phytopathogenic fungi. Due to the extensive and injudicious use of such agrochemicals, massive amounts of toxic fungicide residues can persist in soils for extended durations [6]. Such excess fungicide residues also engender severely toxic effects on crops’ growth and development [7]. The excess use of chemical fungicides also threatens the existence and physiological functions of indigenous beneficial soil microbes [8]. Plant growth-promoting rhizobacteria (PGPR), for example, are a group of free-living bacteria that colonize the rhizosphere and provide benefits to root physiology and growth [9,10]. Fungicides also adversely affect the function and composition of soil enzymes, as well as microbial respiration, biomass production, diversity, and function [11,12]. Among fungicides, hexaconazole (HEXA), for example, has been found to adversely affect the various metabolic activities of plants [13]. Additionally, repeated use of fungicides has led to the emergence of resistance among fungal phytopathogens.

Fungicide residues are taken up by vegetable crops and accumulate in different organs and may be translocated to edible portions [14,15]. Attempts have been made to monitor fungicide residues in vegetables including radish by rapid fingerprinting techniques [16]. However, no comprehensive studies have been performed utilizing the fungicide tolerance potential of PGPR to circumvent fungicide toxicity to radish plants. Thus, there is an urgent need to formulate the strategies to obviate such problems in radish crops. Some PGPR strains manage to survive in soil contaminated with high concentrations of fungicides [17]. In this regard, beneficial soil bacteria, which tolerate high levels of fungicides while retaining the ability to secrete plant growth regulators, have been discovered, characterized and applied to minimize the fungicidal toxicity to agricultural production. For example, pesticide-tolerant microbes including N_2_-fixers such as *Azotobacter* [18], *Azospirillum* [19], *Pseudomonas* [20,21], *Kocuria erythromyxa* [22], *Bacillus* [23], and some phosphate-solubilizing bacteria [24] have been reported to play a crucial role in detoxification of toxic compounds including fungicides, leading ultimately to enhanced vegetable production. In particular, Mohamed and Gomaa [21] reported that pesticide-tolerant PGPR strains enhanced the biological attributes and leaf pigments of radish crops grown under adverse conditions.

PGPR enhance vegetable growth by synthesizing bioactive substances, which are available directly to the plant [25] The PGPR from different rhizospheres share certain common mechanisms of plant growth promotion. The production of biomolecules enhances plant growth directly by supplying nitrogen, phosphorus, and plant hormones, etc. or indirectly by producing hydrogen cyanide (HCN), antibiotics, and siderophores [26]. Root exudates influence rhizospheric colonization by PGPR [27,28]. Other soil organisms also impact the activity of PGPR strains; for example, the interaction of *Pseudomonas trivialis* with earthworms and radish plants enhanced radish growth-promoting activity of this bacterium by producing indole-3-acetic-acid, ACC deaminase and siderophores, and solubilizing insoluble forms of phosphorus [29]. Metal-tolerant PGPR also provide stability towards radish plants. For example, lead (Pb)- and cadmium (Cd)-tolerating *Pseudomonas putida* and *Lysinibacillus varians* moderated the radish growth even in Pb- and Cd-contaminated soils by secreting IAA, siderophores, fixing nitrogen, producing ammonia, and solubilizing phosphorus [30]. Two other PGPR, i.e., *Stenotrophomonas* sp. and *Bacillus* sp., mitigated the nickel toxicity in radish plants and increased plant length, biomass, nitrogen content, and chlorophyll content [31]. Furthermore, individual inoculation of *Pseudomonas putida*, *Azotobacter chroococcum*, and *Lactobacillus* sp. to radish plants increased the salinity tolerance of plants [32]. Besides all these studies, the toxic impacts of fungicides on radish growth and development and whether fungicide-tolerant PGPR could mitigate such fungicidal toxicity or not are underexplored. Therefore, to fill these knowledge gaps, we isolated fungicide-tolerant PGPR and investigated their fungicidal toxicity suppression and plant growth-promoting activities towards white radish cropping.

Considering the importance of radish in the Indian diet, the nutrient storehouse of soils; the deleterious impacts of fungicides on the functional composition of soil microbiota, soil fertility and crop productivity; and the lack of adequate information on fungicidal response to radish, this study was undertaken with the following objectives i.e., to: (i) isolate and identify PGPR isolates from different rhizosphere soils; (ii) evaluate fungicidal tolerance by selected PGPR isolates; (iii) determine the production of growth-regulating bio-stimulants under fungicide pressure; (iv) assess the fungicidal toxicity to *R*. *sativus* under both in vitro bioassays and greenhouse conditions; (v) evaluate the remediation potential of identified PGPR on biochemical attributes of *R*. *sativus*; and (vi) determine the production of stressor molecules (proline and malondialdehyde) in fungicide-treated and PGPR-inoculated plants.

## 2. Materials and Methods

### 2.1. Toxicity of Fungicides to R. sativus In Vitro

#### Seed Germination and Plant Growth

Seeds of *R. sativus* were soaked in deionized water for 24 h. Seeds were disinfected with 3% sodium hypochlorite (NaOCl) solution and carefully rinsed with sterile water. Soft agar (0.7%) plates were supplemented with different concentrations of carbendazim (CBZM) and hexaconazole (HEXA) (details appear in Supplementary Table S1). Soft agar plates without fungicide treatment served as a control. Seeds were placed on agar plates and maintained at room temperature (28 ± 2 °C) for 3–4 days. After 4 days, percent germination, and root and shoot lengths of the plantlets were recorded

### 2.2. Isolation of Rhizobacteria

Rhizosphere soils (sandy clay loam and had organic C 6.2 g kg^−1^, Kjeldahl N 0.75 g kg^−1^, Olsen P 16 mg kg^−1^, pH 7.2 and WHC 0.44 mL g^−1^, cation exchange capacity 11.7 and 5.1 cmol kg^−1^ anion exchange capacity) were collected in proximity to cabbage and mustard plants grown at Aligarh Muslim University (AMU), Aligarh (27°53′ N 78°05′ E 27.88° N 78.08° E), Uttar Pradesh, India. Soil samples were diluted serially (10^−1^–10^−7^) in normal saline solution (NSS) and 100 µL aliquots were uniformly spread on King’s B (g L^−1^: protease peptone 20; glycerol 10; K_2_HPO_4_ 1.5; MgSO_4_ 1.5; agar 20; pH 7.4; HiMedia, Mumbai, India) media. Plates were incubated for 1–2 days at 28 ± 2 °C. After incubation, bacterial colonies appearing on King’s B medium that fluoresced under a UV transilluminator were concluded to *Pseudomonas*. Isolates were collected and purified by streaking on King’s B medium three times. The selected isolates were subsequently tested for morphological and biochemical characteristics. (see Supplementary Methods).

### 2.3. Selection of Fungicide-Tolerant PGPR

The selected PGPR isolates were further evaluated for tolerance to fungicides. Isolates were cultured in minimal salt agar (MS) medium (g L^−1^: K_2_HPO_4_ 7.0; KH_2_PO_4_ 2.0; Na_3_C_6_H_5_O_7_ 0.2; MgSO_4_ 0.1; (NH_4_)_2_SO_4_ 1.0; agar 20; pH 7.4; HiMedia, Mumbai, India) supplemented with varying concentrations (0, 100, 200, 400, 800, 1600, and 3200 µg mL^−1^) of carbendazim and hexaconazole (see Supplementary Table S1) followed by incubation at 28 ± 2 °C in a shaking incubator (150 rpm) for 2–3 days. After incubation, the metabolically active cells were counted using the spread plate viable count method. The isolates exhibiting tolerance to high concentrations of fungicides were referred to as fungicide-tolerant PGPR isolates.

### 2.4. Morphological, Biochemical and Molecular Identification of PGPR Isolates

Fungicide-tolerant PGPR isolates (AZ2 and PS3) were further tested for morphological features using Gram’s staining (see Section 2.4.1 in Supplementary Materials) and characterized biochemically using Bergey’s Manual of Determinative Bacteriology (see Sections 2.4.2 to 2.4.10 in Supplementary Materials). For identification of isolates to the genus level, 16S rRNA sequencing was performed [33] (see Supplementary Materials, Dtailed methods of DNA isolation, PCR, sequencing).

### 2.5. Production of Plant Growth-Promoting Substances Under Fungicidal Stress

#### 2.5.1. Indole-3-Acetic-Acid (IAA) and Siderophore Production

IAA synthesized by tolerant PGPR isolates was determined quantitatively [34]. Ten millilters of Luria Bertani (LB) broth (HiMedia, Mumbai, India) containing 100 µg mL^−1^ of tryptophan and supplemented with 0 (control), 1X (500 μg mL^−1^), 2X (1000 μg mL^−1^), and 3X (1500 μg mL^−1^) concentrations each of CBZM and HEXA were inoculated with 100 µL of freshly prepared inoculum (for details, see Supplementary Information).

Siderophore produced by both PGPR isolates were detected by the FeCl_3_ test [35]. A 10 mL aliquot of autoclaved liquid nutrient broth (NB) amended with varying doses of CBZM and HEXA was inoculated with PGPR isolates and incubated at 28 ± 2 °C for 4 days. Following incubation, bacterial cells were centrifuged at 7000× *g*. From the supernatant, 1.0 mL was removed and mixed with 1.0 mL of FeCl_3_ (2%; Sisco Research Laboratory Pvt. Ltd., Mumbai, India) in a culture tube, and absorbance was recorded following the method of Shahid and Khan [36].

#### 2.5.2. Phosphate Solubilization

The quantitative and qualitative determination of phosphate-solubilizing activity (PSA) of PGPR isolates was performed utilizing Pikovskaya’s (PKV) agar procured from HiMedia, Mumbai, India [g L^−1^: glucose 10; Ca_3_(PO_4_)_2_ 5; (NH_4_)_2_SO_4_ 0.5; NaCl 0.2; MgSO_4_⋅7H_2_O 0.1; KCl 0.1; yeast extract 0.5; MnSO_4_ and FeSO_4_ trace; agar 15; pH 7.0] (both solid and liquid) medium amended with three concentrations of test fungicides. The quantity of solubilized phosphate in liquid media was assessed by chlorostannous (SnCl_4_; Thermo Fisher Scientific, Mumbai, India) reduced molybdophosphoric acid (Thermo Fisher Scientific, Mumbai, India) blue method of Jackson [37].

#### 2.5.3. Hydrogen Cyanide (HCN) and Ammonia Production

To evaluate the production of cyanogenic cyanogenic compounds by fungicide-tolerant PGPR, hydrogen cyanide (HCN) induction medium (King’s B + 4.4 g glycine L^−1^) was autoclaved and different concentrations of fungicide were added. Test PGPR were densely streaked on plates. Discs of filter paper were imbibed in 0.5% picric acid solutions (Thermo Fisher Scientific, Mumbai, India) and placed on the upper side of the plate, wrapped and incubated at 28 ± 2 °C [38]. Color change of the filter paper was recorded. For ammonia production, PGPR isolates were inoculated in autoclaved peptone water, amended with varying doses of fungicide and kept at 28 ± 2 °C for 3–4 days. One milliliter of Nessler’s reagent was subsequently added drop-wise to a culture tube, and color changes in the liquid broth were recorded [39].

### 2.6. Assessment of Fungicidal Toxicity to PGPR Isolates

#### 2.6.1. Morphological Distortion Induced by Fungicides Observed by Scanning Electron Microscope (SEM)

To assess the effect of the fungicides on surface morphology of both test PGPR isolates (AZ2 and PS3), scanning electron microscopy was performed. Both isolates were cultured in liquid NB broth amended with 1500 µg mL^−1^ each of the fungicides and kept at 28 ± 2 °C for 24 h. Cultures raised in fungicide-free broth were established for comparison. The method of Shahid et al. [40] was followed for preparation of cultures for SEM examination (JSM 6510 LV, JEOL, Tokyo, Japan) (See Supplementary Method).

#### 2.6.2. Assessment of Membrane Integrity by Confocal Laser Scanning Microscope (CLSM)

Fungicide-induced changes in integrity of bacterial membrane were assessed using CLSM following the method of Shahid and Khan [41]. A total of 200 µL fungicide-treated or untreated suspensions of freshly grown bacteria and 10 µL propidium iodide [PI: 50 μg mL^−1^ prepared in phosphate buffer saline (PBS)] dye was mixed. Then solution was kept at 28 °C for 10–15 min, following which cells were harvested by centrifugation at 5000× *g* for 10 min. The cell pellets were re-dissolved in PBS (500 µL), then 10 µL of this was smeared on a glass cover slip, and examined under CLSM (LSM-780, Leica Confocal microscope, Zeiss, Oberkochen, Germany).

### 2.7. Crop-Based Experiments

#### 2.7.1. Fungicide Application, Seed Treatment and Plant Culturing

Healthy seeds of radish (*R*. *sativus*) were surface sterilized as mentioned above. Commercial grade fungicides, carbendazim [recommended dose: 1X (2 mg kg^−1^), 2X (5 mg kg^−1^) and 3X (10 mg kg^−1^)] and hexaconazole [recommended dose: 1X (2 mg kg^−1^), 2X (5 mg kg^−1^) and 3X (10 mg kg^−1^)] were applied to moist soils at least 1 day before sowing seeds. The physiochemical properties of the soil are shown in Supplementary Table S2. The soil was placed into 20 × 24 cm clay pots with approximately 5 kg soil per pot. The disinfected radish seeds were coated with individually grown cultures of *Pseudomonas* sp. PS3 (grown for 48 h) and *Pseudomonas* sp. AZ2 (grown for 72 h) by immersing them in liquid culture medium for 2 h using 10% gum arabic as an adhesive to achieve 1 × 10^8^ cells seed^−1^. Un-inoculated sterilized seeds submerged in sterile water were used as control. Seeds (*n* = 10) were sown in respective earthen pots containing 5 kg of soil. Sowing was carried out and germination was recorded 7 days after sowing (DAS). Two controls were run in parallel; one was uninoculated and untreated (without bacteria and without fungicide) and the second was inoculated (bacteria but no fungicide). Pots without fungicide treatment but bacterized (i.e., coated with PGPR isolates) radish seeds also served as a control treatment for comparison.

Exposure at each fungicide concentration was replicated thrice and pots were arranged in a completely randomized block design. After germination, seedlings were thinned and two uniform healthy seedlings of radish were maintained in each pot, 15 days after emergence (DAE). Pots were kept in an open field condition (9 h light/15 h dark cycle) and watered regularly using tap water. The crop experiments were carried out for 2 years to achieve consistency and reproducibility in results.

#### 2.7.2. Measurement of Growth Attributes

Fungicide-treated and bacterized radish plants were removed from pots at 60 DAS and the height and weight of roots and shoots were measured. Dry matter was also measured. For determination of root and shoot biomass (dry weight), samples were removed carefully from soil and washed repeatedly with tap water; after drying on tissue papers, roots and shoots were dried at 80 °C in a ventilated oven (Yorco, York Scientific Industries, Pvt. Ltd. India) for 30 min. and then further dried to constant weight at 60 °C for 48 h. After drying, root, shoot, and whole plant biomass (g plant^−1^) were weighed using an electronic scale balance (BL-220 H, Shimadzu, Japan).

#### 2.7.3. Effect of PGPR Inoculants on Photosynthetic Pigments

The production of chlorophyll molecules (total chlorophyll) and carotenoids was evaluated in fresh leaves of control and plants treated with three different concentrations (1X, 2X, and 3X) of fungicides and bacterized with fungicide-tolerant PGPR isolates following the methods of Shahid et al. [42]. See Supplementary Methods in the supplementary materials.

#### 2.7.4. Determination of Total Soluble Protein

Protein content in fungicide-treated/untreated and PGPR-inoculated radish leaves were determined [43] using bovine serum albumin (BSA; HiMedia, Mumbai, India) as standard. For the assay, 1.0 g of fresh leaf was crushed in 3.0 mL of 50 mM phosphate buffer (pH = 7.8) containing 1 mM EDTA (HiMedia, Mumbai, India) and 2% *w/v* polyvinyl pyrrolidone (PVP; HiMedia, Mumbai, India). The extract was centrifuged at 10,000 ×*g* and set aside for 10 min at 4 °C. For protein estimation, 0.2 mL supernatant was placed in a glass test tube and the volume made up to 1.0 mL with double distilled water (DDW). To this, 4.5 mL of copper solution was added and kept for 10 min. Thereafter, 0.5 mL of Folin’s reagent (Thermo Fischer Scientific) was added to each tube and incubated for 30 min for color development. Protein content was recorded by measuring the absorbance at 660 nm against the calibration curve of BSA.

### 2.8. Assessment of Stressor Molecules and Antioxidant Enzymes in Radish

#### 2.8.1. Proline Estimation

The free proline content in PGPR-inoculated leaves of radish plants cultivated with and without fungicide amendment was assayed according to Bates et al. [44] (see Supplementary Methods).

#### 2.8.2. Lipid Peroxidation (MDA content)

Lipid peroxidation in radish tissue was evaluated via measurement of malondialdehyde (MDA) production. Adduct formation (MDA-TBA2) between thiobarbituric acid (TBA) and MDA was recorded and measured spectrophotometrically (Shimadzu, 2600, Tokyo, Japan) in fungicide-treated radish foliage (Heath and Packer, 1968). Fresh foliage (500 mg) was homogenized with 10 mL trichloroacetic acid (TCA; 5% *w*/*v*; HiMedia, Mumbai, India) in an ice bath followed by centrifugation (12,000× *g*) at 4 °C for 20 min. Equal volumes of resulting supernatant and thiobarbituric acid (TBA; 0.67% *w*/*v*; HiMedia, Mumbai, India) were mixed in an acid-rinsed glass tube followed by heating at 100 °C in a water bath for 30 min, and then placed on an ice bath to terminate the reaction. After centrifugation (10,000× *g*) at 4 °C for 10 min, the optical density of the supernatant was recorded at three wavelengths (λ) 450, 532, and 600. The MDA levels were calculated using the following equation [45] and the molar extinction coefficient of 155 mM^−1^ cm^−1^:

MDA level (µmol mL^−1^) = 6.45 × (Absorbance_532 nm_ − Absorbance_600 nm_) − 0.56 × Absorbance_450 nm_ (1)

#### 2.8.3. Extraction and Determination of Antioxidant Enzymes

Levels of catalase (CAT), glutathione reductase (GR), and ascorbate peroxidase (APX) in fungicide-treated and PGPR-inoculated leaves of radish were determined (See Supplementary Methods in the supplementary materials).

### 2.9. Statistical Analysis

Data were statistically analyzed using Sigma Plot 12.0 and Minitab17 software. Tests included two-way analysis of variance (ANOVA) followed by post-hoc least significant difference (LSD). Student’s *t*-test was used where applicable.

## 3. Results and Discussion

### 3.1. Germination, Vigor Index and Biological Attributes of R. Sativus Under Fungicide-Stressed Conditions

**Figure 1:**
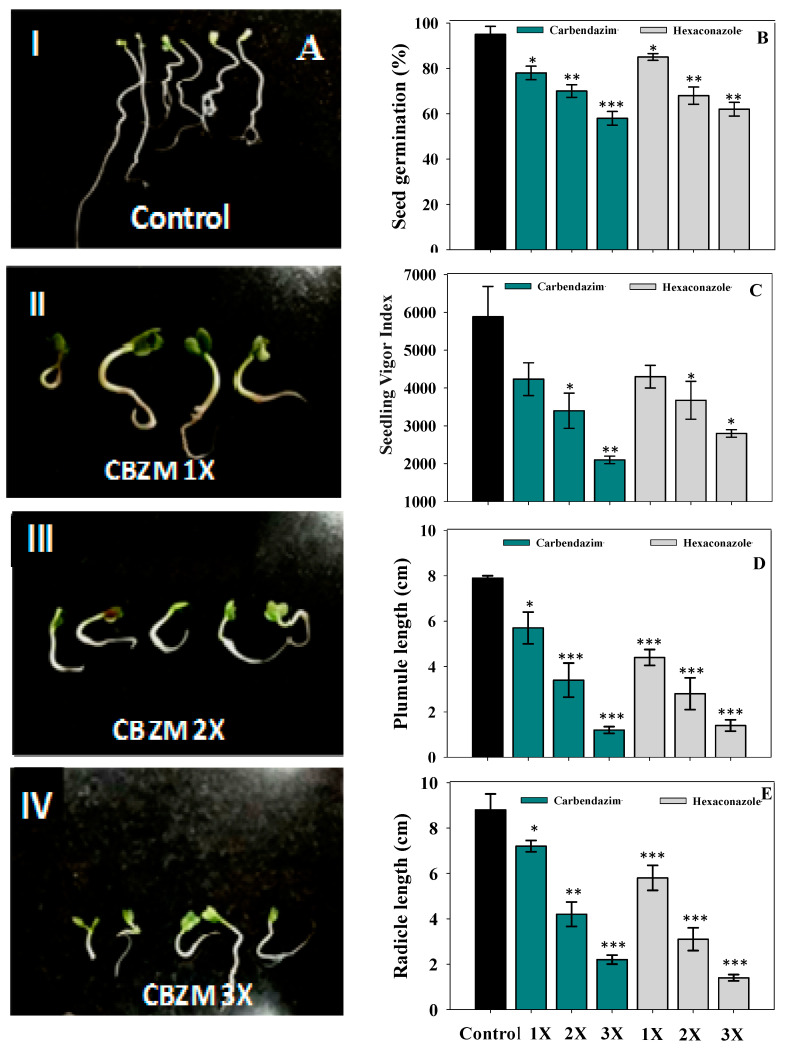
Effect of fungicide on seed germination efficiency and biological attributes of *R. sativus*: (**A**) Seeds germinated on agar untreated and treated with 1X, 2X and 3X concentrations of carbendazim (CBZM); (**B**) Effect of CBZM and hexaconazole (HEXA) on percent germination; (**C**) seedling vigor index; (**D**) plumule length; and (**E**) radicle length. In this and succeeding figures, histograms represent the mean value of three independent replicates (*n* = 3) and error bars represent standard deviation (S.D). The asterisks *, ** and *** denote statistical significance at *p* < 0.05, *p* < 0.005 and *p* < 0.001, respectively computed by Student’s *t*-test. 1X, 2X and 3X denote the normal, two times and three times greater concentrations of fungicide.

Under fungicide-free conditions (control), percent seed germination, seedling vigor index (SVI), radicle length (RL), and plumule length (PL) were 95%, 5645 SVI, 8.5 cm and 8 cm, respectively (Figure 1). The fungicides inhibited all parameters; percent germination, SVI, RL, and PL were significantly reduced by 38% (*p* < 0.001), 64% (*p* < 0.005), 81% (*p* < 0.001), and 76% (*p* < 0.001), respectively, at 3X dose of CBZM compared to control (Figure 1). The toxic effects of different concentrations of the fungicide kitazin on germination efficiency, vigor index and length of pea plants under in vitro conditions has also been reported (Shahid et al., 2019) [46].

### 3.2. Biochemical Characterization, Identification and Fungicide Tolerance

**Figure 2:**
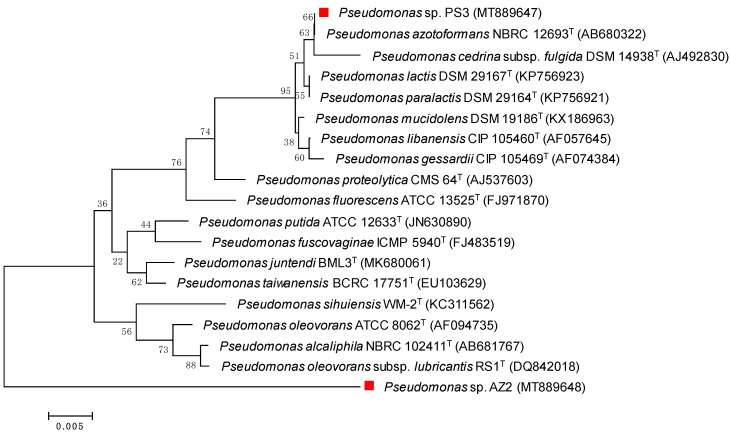
Unrooted neighbour-joined phylogenetic tree of two plant growth-promoting rhizobacteria (PGPR) Pseudomonas sp. PS3 and Pseudomonas sp. AZ2 isolated from cabbage and mustard rhizospheres. The tree was constructed based on 16S rRNA partial gene sequence of selected PGPR (marked with red square) and closely related phylogenetic species (type cultures) derived using NCBI BLAST search tool. Sequences were aligned using Clustal W sequence alignment tool in MEGA 7.0 software. The GenBank accession numbers of isolates and closely related species are presented in parenthesis. Bootstrap percentage values as obtained from 1000 replications of the data set are given at the tree’s nodes. The scale bar corresponds to the mean number of nucleotide substitutions per site.

The morphological, biochemical and cultural characteristics of selected PGPR isolates recovered from rhizosphere soils of cabbage (AZ2), and mustard (PS3) varied significantly (Table 1). Microscopic analysis revealed that both isolates were Gram-negative short rods. Based on biochemical reactions, the AZ2 isolate showed positive reactions towards citrate utilization, indole production, methyl red test, oxidase test, Voges Proskauer test, sucrose and mannitol utilization, and starch and gelatin hydrolysis. Isolate PS3 exhibited a positive response to citrate utilization, methyl red, nitrate reduction, oxidase, starch, and gelatin. Based on these biochemical features, both isolates were further identified to the genus level by 16S rRNA partial gene sequencing. By comparing 16S rRNA sequences provided by Macrogen and data available in the NCBI data bank and LPSN web portal, both isolates represent novel species: isolate PS3 showed maximum base sequence similarity (>99.77%) to type strain *Pseudomonas lactis* DSM 29167*^T^* (Accession number KP756923), whereas isolate AZ2 was closely (>94.87%) related to type strain *Pseudomonas sihuiensis* KCTC 32246*^T^* (Accession number KC311562) (Figure 2). Based on these data, isolates were identified as Pseudomonas spp. Hence, there could be minimal differences in *Pseudomonas* species isolated in this study and reported elsewhere in other studies. Various workers have also isolated and characterized the PGPR isolates by 16S rRNA partial gene sequence analysis as well as other state-of-the-art molecular tools [47,48].

The bacterial isolates grown on fungicide-supplemented MSA plates exhibited a significant tolerance to fungicides. Among all the 15 rhizobacterial isolates, *Pseudomonas* sp. AZ2 survived up to 2400 and 1600 µg mL^−1^ each of CBZM and HEXA, respectively, whereas *Pseudomonas* sp. PS3 tolerated 2400 and 3200 µg mL^−1^ each of CBZM and HEXA, respectively. Tolerance to pesticides including fungicides is considered a distinguishing feature among soil-inhabiting microorganisms, which are directed by unique physiological and genetic characteristics. It is likely that microorganisms that tolerate excessively high doses of fungicides are degrading them as well. Researchers have isolated pesticide-tolerant rhizobacteria from rhizospheres of different vegetable crops [49,50,51]. Some Gram-negative bacteria have been reported to withstand high levels of fungicides; for example, *Pseudomonas putida* PS 9 isolated from the mustard rhizosphere exhibited variable tolerance to four fungicides at concentrations ranging from 1400–3200 μg mL^−1^ [52]. Similarly, a *Pseudomonas* strain isolated from the rhizosphere of *Brassica compestris* solubilized an insoluble form of phosphorus and produced IAA, HCN, siderophores, NH_3_, and exopolysachharides, thus enhancing overall growth of *Vigna radiata* in fungicide-amended soils [53].

### 3.3. Plant Growth-Promoting Activities of PGPR Isolates under Fungicide-Stress

#### 3.3.1. IAA and Siderophores

Generally, IAA secreted by both bacterial isolates decreased with increasing concentration of fungicide (Table 2). In fungicide-free media, *Pseudomonas* sp. PS3 and *Pseudomonas* sp. AZ2 produced substantial IAA (61.3 and 39.5 μg mL^−1^, respectively), which declined with increasing fungicide concentration. A maximum reduction of 65% and 52% in the synthesis of IAA was recorded when isolates AZ2 and PS3 were grown in LB broth supplemented with 3X (1500 μg mL^−1^) of carbendazim. Additionally, 1500 μg HEXA mL^−1^ reduced the synthesis of IAA by 57% (for AZ2) and 59% (for PS3) compared to IAA synthesized in the control. Reduction in bacterial synthesis of IAA under pesticide stress has been reported by others (Shahid et al., 2019; Park et al., 2017). Secretion of IAA by fungicide-tolerant PGPR isolates at high levels of fungicides is a promising feature; such PGPR, when employed in harsh environments, are more likely to produce phytohormones such as IAA, making this crucial growth-augmenting hormone accessible to plants even under high levels of fungicide [54,55].

Both isolates (AZ2 and PS3) produced siderophores in liquid media with and without the amendment of fungicides (Table 2). However, the 3X concentration of both fungicides was inhibitory to siderophore production. Siderophores are low-molecular weight chelating complexes released by soil microbial consortia to deliver iron to plants under Fe-deficient conditions [56]. Two major forms of insoluble iron occur in soil: (i) hydroxides and (ii) oxyhydroxides, both of which are unavailable to rhizobacteria [57]. Hence, release of siderophores under Fe-limited conditions could be useful, as siderophore-producing PGPR isolates may be applied for the bio-management of plant pathogens [58,59]. Production of siderophores by soil microorganisms in stressed environments has previously been reported [41,60,61].

#### 3.3.2. Phosphate Solubilization, Cyanogenic Compounds and Ammonia Production

Isolates AZ2 and PS3 solubilized 26.2 and 36 μg mL^−1^ phosphates, which decreased with increased fungicide concentration (Table 2). In the case of *Pseudomonas* sp. AZ2, the quantity of solubilized phosphate was reduced from 26.2 μg mL^−1^ to 25.8, 20, and 14.4 μg mL^−1^ at 500 (1X), 1000 (2X) and 1500 (3X) μg mL^−1^ of carbendazim, respectively. Similarly, at 1500 µg HEXA mL^−1^, the phosphate-solubilization activity of *Pseudomonas* sp. AZ2 decreased by 46% compared to control. The 1500 µg mL^−1^ rate of carbendazim and hexaconazole reduced phosphate-solubilization activity of *Pseudomonas* sp. PS3 by 58% and 67%, respectively, compared to the control. The phosphate-solubilizing property of rhizobacterial isolates is recognized to be variable due to differences in ability to release several organic acids (acetic, oxalic, citric, malic, maleic, succinic, gluconic, α-2 keto gluconic) that reduce the pH of the medium [62]. A similar reduction in phosphate-solubilization potential of *Azotobacter vinelandii* under different groups of pesticides has been reported [40].

Cyanogenic compounds are secreted by soil microbiota as a secondary metabolite [63]. These compounds are synthesized by bacteria, fungi and plants via cyanogenesis [64]. Isolates AZ2 and PS3 showed a positive reaction toward hydrogen cyanide activity. Both isolates showed a positive reaction to HCN production even at high fungicide concentration. Similarly, isolates AZ2 and PS3 exhibited positive response for ammonia production at all test concentrations of fungicides. Different species of *Bacillus* [65], *Pseudomonas* [66], *Aeromonas* [67], and *Alcaligenes* [68] are reported to produce cyanogenic compounds under adverse environments.

### 3.4. Assessment of Cell Morphology and Permeability

**Figure 3:**
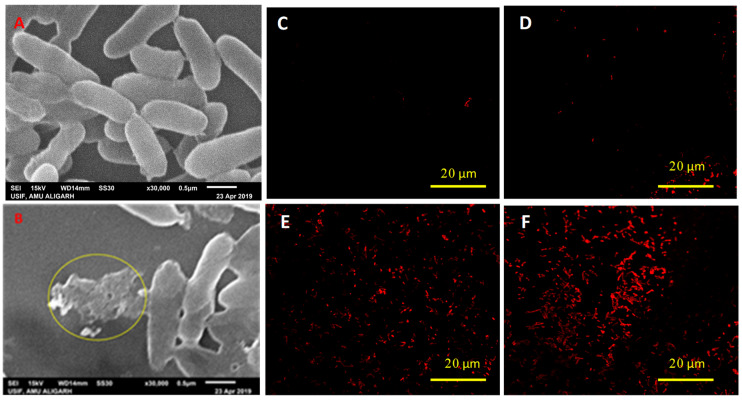
Scanning electron microscopic images of *Pseudomonas* sp. PS3. (**A**) untreated/control cells; and (**B**) cells treated with 1000 μg hexaconazole (HEXA) mL^−1^. The yellow circle shows broken/damaged cells after exposure to fungicide. (**C**) CLSM images of fungicide-untreated cells of *Pseudomonas* sp. PS3; (**D**) cells treated with 500, (**E**) 1000, and (**F**) 1500 μg HEXA mL^−1^. The red-colored rods represent dead cells with increased fungicide concentration.

**Figure 4:**
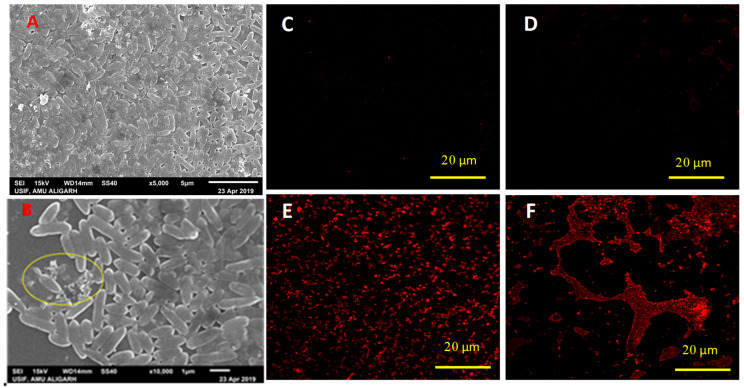
Scanning electron microscopic images of *Pseudomonas* sp. AZ2. (**A**) untreated/control cells; (**B**) cells treated with 1000 μg hexaconazole (HEXA) mL^−1^. The yellow circle shows broken/damaged cells after exposure to fungicide. (**C**) CLSM images of fungicide-untreated cells of *Pseudomonas* sp. AZ2; (**D**) treated with 500, (**E**) 1000, and (**F**) 1500 μg HEXA mL^−1^. The red rods represent dead cells with increased fungicide concentration.

Cells of bacterial isolates PS3 and AZ2 exposed to 1000 μg mL^−1^ of both fungicides experienced considerable impacts on cell shape and surface morphology, i.e., distortion of cell shape, increased cell length, and multiple cracks on the cell envelope (Figure 3B and Figure 4B). In contrast, untreated cells possessed intact surfaces and structure (Figure 3A and Figure 4A). Marked changes in cell morphology under the stress xenobiotics like fungicides are accepted parameters for assessment of bacterial survival [69,70]. The microscopic data corroborates with similar morphological transformations reported for other bacterial species [33,40,71].

The extent of damage caused by fungicides to bacterial cells was assessed in terms of membrane permeability measured by staining the cells with a fluorescent dye propidium iodide (PI). PI has an inherent property of binding to DNA only if membrane permeability is compromised. CLSM analysis of HEXA (500–1500 μg mL^−1^)-treated cells of *Pseudomonas* sp. PS3 (Figure 3D–F) and Pseudomonas sp. AZ2 (Figure 4D–F) suggest membrane destruction by fungicides. A large number of cells treated with fungicide were observed to emit fluorescence of PI at a rate greater than those of untreated cells. No red color was observed in CLSM images of fungicide-untreated cells (Figure 3C and Figure 4C); this was concluded to indicate cell damage. Cell membranes act as selectively permeable barriers for a variety of molecules; however, if permeability increases, cells may take up excessive quantities of materials present in their surroundings [72]. Therefore, stressor molecules including fungicides gain access to intracellular locations and exert toxic impacts mostly in the form of intracellular oxidative stress. To differentiate between viable and dead cell fluorescence, cells were stained with PI. Metabolically active cells with intact cell membranes do not selectively take up PI; rather, it is taken up by membrane-compromised cells [73]. Similar observations have been made for viable and dead cells of Bacillus subtilis in a pesticide-contaminated environment [36].

### 3.5. Radish-Fungicide-PGPR Interactions

#### 3.5.1. Performance of *R. sativus* under Fungicide-Stressed Conditions

##### Plant Growth and Elongation

Radish root and shoot lengths treated with fungicides varied at 60 DAS; generally, a progressive and dose-dependent decline was observed for both (Supplementary Tables S3 and S4). HEXA at 3X (10 mg kg^−1^ soil) dose exhibited severe toxic effects and decreased root (RL), shoot (SL) and whole plant (WPL) length by 47.5%, 47% and 47.7%, respectively, over the untreated control. Similarly, CBZM at 10 mg kg^−1^ soil (3X) reduced the length of roots, shoots and whole plant by 46%, 45% and 53.8% (*p* < 0.005), respectively.

**Figure 5:**
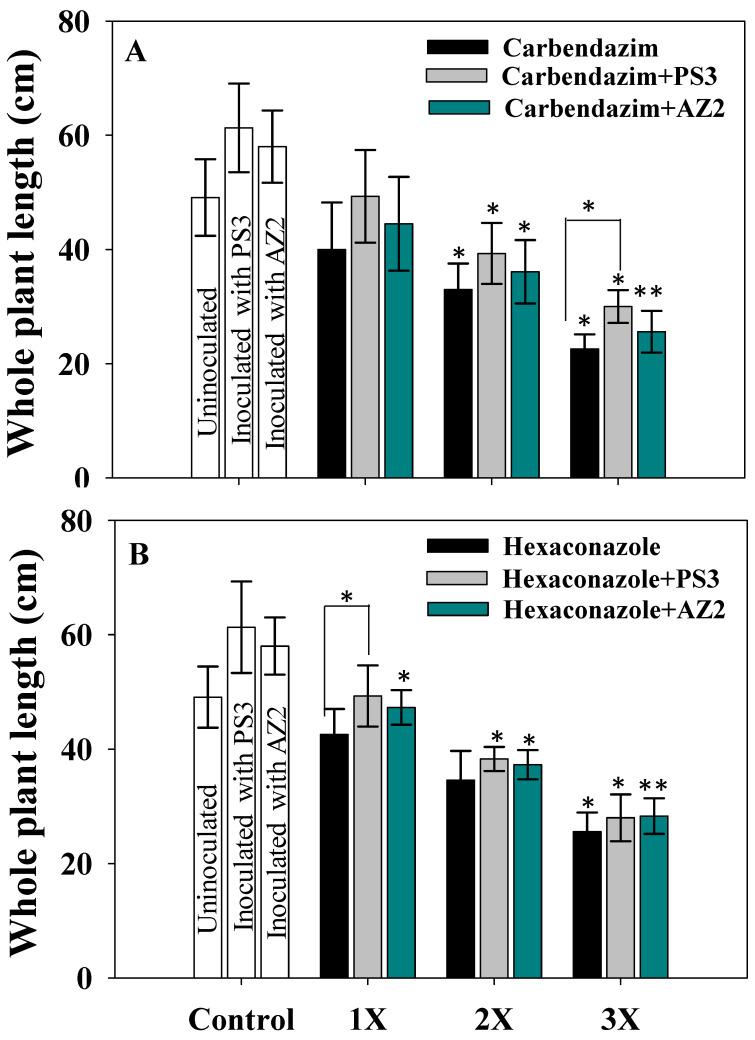
Bio-inoculation effect of *Pseudomonas* sp. PS3 and *Pseudomonas* sp. AZ2 on whole plant length (cm) of *R. sativus* plants grown in soil treated with varying concentrations of carbendazim (**A**) and hexaconazole (**B**). In these figures, histograms represent the mean value of three independent replicates (*n* = 3) and error bars represent S.D. The asterisks * and ** denote statistical significance at *p* < 0.05 and *p* < 0.005, respectively computed by Student’s *t*-test. 1X, 2X and 3X denote the normal, two times and three times greater concentrations of fungicide.

PGPR (*Pseudomonas* sp. AZ2 and *Pseudomonas* sp. PS3)-inoculated radish reduced the toxic effects of the fungicides and improved overall plant growth. These results were due to fungicide detoxification/degradation by the isolates. *Pseudomonas* sp. PS3 improved RL, SL and WPL by 15%, 18% and 24.6%, respectively, at 10 mg CBZM kg^−1^ (3X dose) relative to uninoculated but identical CBZM dosage (Supplementary Table S4 and Figure 5A). *Pseudomonas* sp. AZ2 increased the RL, SL and WPL by 37%, 46% and 9.5% (*p* < 0.005), respectively, in the presence of 10 mg HEXA kg^−1^ over the un-inoculated but HEXA-treated plants (Supplementary Table S3 and Figure 5B). Similar to these findings, herbicide-induced decline in growth of greengram tissue and ameliorative effects of stress-tolerant PGPR under greenhouse conditions was reported by Shahid et al. [42].

##### Dry Biomass Accumulation

**Figure 6:**
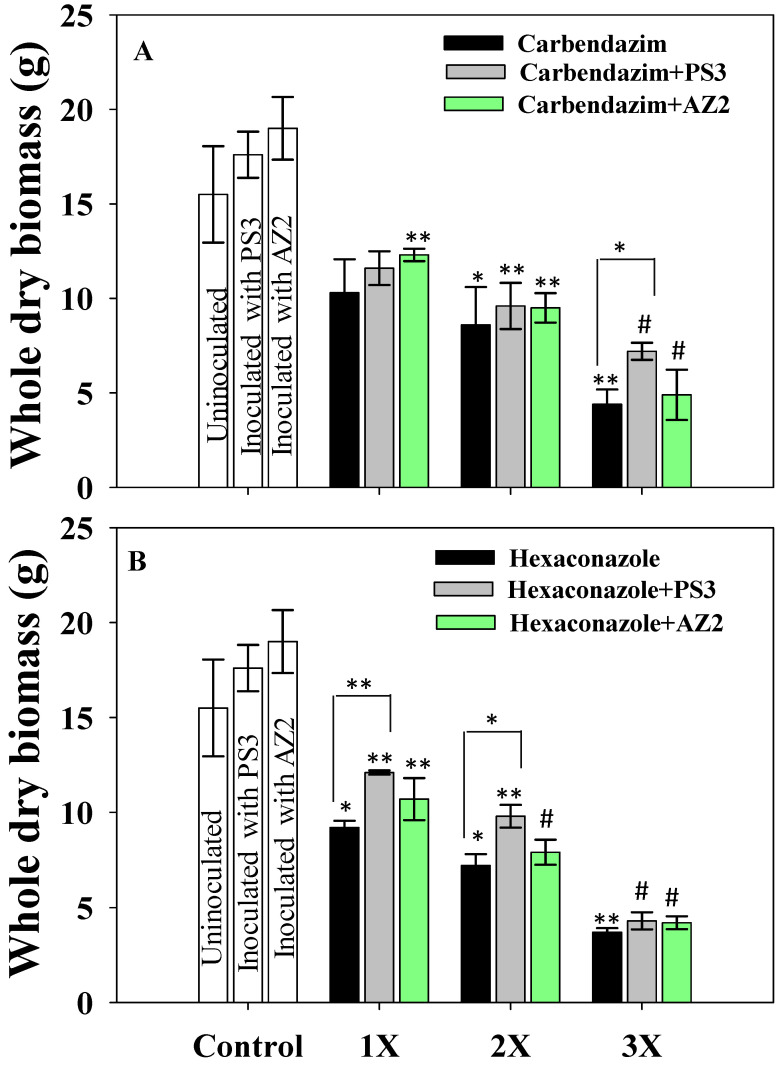
Bio-inoculation effect of *Pseudomonas* sp. PS3 and *Pseudomonas* sp. AZ2 on whole dry biomass (g) of *R. sativus* plants grown in soils treated with varying concentrations of fungicide carbendazim (**A**) and Hexaconazole (**B**). In this figures, histograms represent the mean value of three independent replicates (*n* = 3) and error bars represent S.D. The asterisks *, ** and # denote statistical significance at *p* < 0.05, *p* < 0.005 and *p* < 0.001, respectively computed by Student’s *t*-test. 1X, 2X and 3X denote the normal, two times and three times greater concentrations of fungicide.

The phytotoxic effects of different doses of fungicides on dry matter accumulation by *R. sativus* plants diverged noticeably (Supplementary Tables S3 and S4). Fungicide-driven toxicity to dry biomass production increased progressively with increasing concentration. The 5 mg CBZM kg^−1^ (2X concentration) dosage reduced root, shoot and total dry biomass of radish by 57%, 38% and 44.5%, respectively over control (Supplementary Tables S3 and S4 and Figure 6A). In contrast, a gradual increase in dry biomass of radish roots and shoots inoculated with PGPR while simultaneously treated with varying doses of fungicide was observed. The maximum positive effect of PGPR was recorded at the lower fungicide levels. *Pseudomonas* sp. PS3 maximally increased radish total dry biomass by 33% when applied to soil treated with 2 mg CBZM kg^−1^ (1X concentration) compared to dry biomass of uninoculated but treated with the same rate of CBZM (Figure 6A). Similarly, *Pseudomonas* sp. AZ2 increased dry biomass of roots and shoots by 8.8 and 9.7%, respectively, when grown in soil treated with 2 mg kg^−1^ soil each of CBZM and HEXA, respectively, compared to uninoculated but treated with the same fungicide concentration (Figure 6A,B).

##### Total Chlorophyll and Carotenoid Content

**Figure 7:**
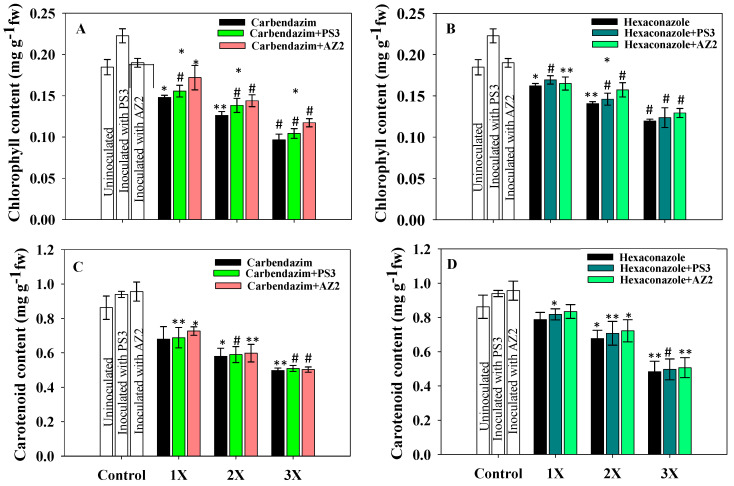
Effect of inoculation of *Pseudomonas* sp. PS3 and *Pseudomonas* sp. AZ2 on total chlorophyll content mg g^−1^ fresh weight (fw) in foliage of *R. sativus* treated with varying concentrations of carbendazim (**A**) and hexaconazole (**B**). Panels (**C**,**D**) represent the impact of *Pseudomonas* sp. PS3 and *Pseudomonas* sp. AZ2 on carotenoid content extracted from radish foliage. In this figure, histograms represent the mean value of three independent replicates (*n* = 3) and error bars represent S.D. The asterisks *, ** and # denote statistical significance at *p* < 0.05, *p* < 0.005 and *p* < 0.001, respectively computed by Student’s *t*-test. 1X, 2X and 3X denote the normal, two times and three times greater concentrations of fungicide.

Leaf pigments (chlorophyll and carotenoid) extracted from foliage of PGPR- and fungicide-supplemented radish plants uprooted at 60 DAS declined consistently with increasing fungicide dose (Figure 7). Lesser toxic impacts on chlorophyll synthesis were detected at the lower fungicide concentration compared to those recorded for the 2X and 3X doses. CBZM at 2 mg kg^−1^ soil decreased chlorophyll content by 17% (*p* < 0.05) and 33%, respectively, over uninoculated/untreated radish plants. The 3X dose of fungicides had a severe toxic impact on chlorophyll synthesis; at the CBZM 3X dose rate, total chlorophyll and carotenoid contents decreased by 50% (*p* < 0.001) and 41% (*p* < 0.005), respectively, over the untreated control. Additionally, 10 mg HEXA kg^−1^ decreased total chlorophyll and carotenoid contents of fresh foliage by 37% (*p* < 0.001) and 47% (*p* < 0.005), respectively, compared to the untreated control.

A gradual increase in chlorophyll and carotenoid contents was observed in PGPR-inoculated and fungicide-treated foliage compared to uninoculated plants. *Pseudomonas* sp. PS3 increased chlorophyll content by 9.6% (*p* < 0.001) and 5.3% (*p* < 0.001) and carotenoid content by 2.5% (*p* < 0.005) and 4.5% (*p* < 0.05) at 2 mg kg^−1^ soil each of carbendazim (Figure 7A) and hexaconazole (Figure 7B), respectively. *Pseudomonas* sp. AZ2, when inoculated with 2 mg CBZM kg^−1^ soil, increased chlorophyll (Figure 7A) and carotenoid contents (Figure 7C) by 17.6% (*p* < 0.05) and 7.7% (*p* < 0.05) over non-PGPR and supplemented with an identical dose of fungicide. Yildrim et al. [22] reported that chlorophyll content in fresh foliage of *Raphanus sativus* was negatively affected under stressed conditions; however, chlorophyll content increased following inoculation of PGPR strains *Staphylococcus kloosii* EY37 and *Kocuria erythromyxa* EY43.

### 3.6. Protein Content

**Figure 8:**
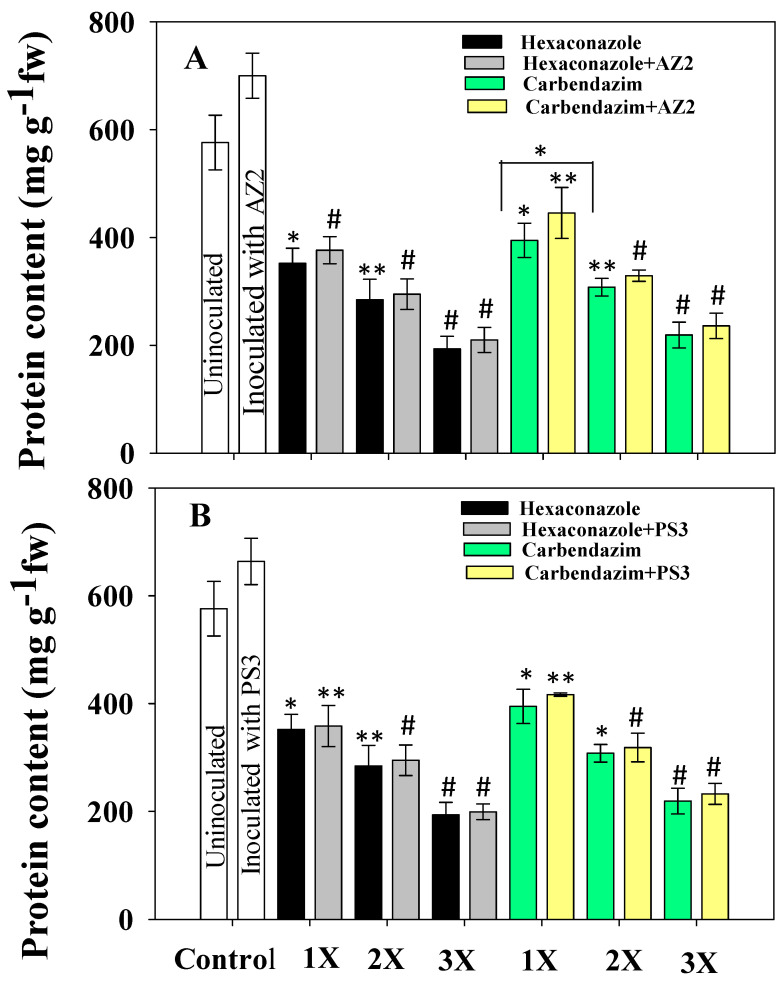
Protein content mg g^−1^ fresh weight (fw) in fresh *R. sativus* grown with varying concentrations of fungicides carbendazim and hexaconazole, and inoculated with *Pseudomonas* sp. AZ2 (**A**) and *Pseudomonas* sp. PS3 (**B**). In this figure, histograms represent the mean value of three independent replicates (*n* = 3) and error bars represent S.D. The asterisks *, ** and # denote statistical significance at *p* < 0.05, *p* < 0.005 and *p* < 0.001, respectively computed by Student’s *t*-test. 1X, 2X and 3X denote the normal, two times and three times greater concentrations of fungicide.

Depending on the level of stressor molecules, total soluble protein (TSP) of the plant may either decrease or increase. The TSP content in foliage of radish plants dosed with fungicides decreased substantially (Figure 8). In the control (uninoculated) treatment, 576 mg g^−1^ fresh weight total soluble protein accumulated in radish leaves, which decreased with increasing fungicide concentration. The 3X dose of HEXA maximally (66%) and significantly (*p* < 0.001) decreased TSP compared to the untreated control. Inoculation of PGPR isolates substantially increased TSP level. *Pseudomonas* sp. AZ2 significantly (*p* < 0.001) increased TSP content of radish from 394 to 445 mg g^−1^ fresh weight (11.5%) at the 1X dose of CBZM (Figure 8A). However, *Pseudomonas* sp. PS3 imparted only marginal improvement in TSP of radish when applied to soil treated with the different doses of fungicide (Figure 8B). The improved TSP content of *R. sativus* plants inoculated with fungicide-tolerant PGPR isolates and supplemented with variable doses of fungicides may be due to the fungicide-degrading potential of the bacterial isolates.

### 3.7. Proline Accumulation

**Figure 9:**
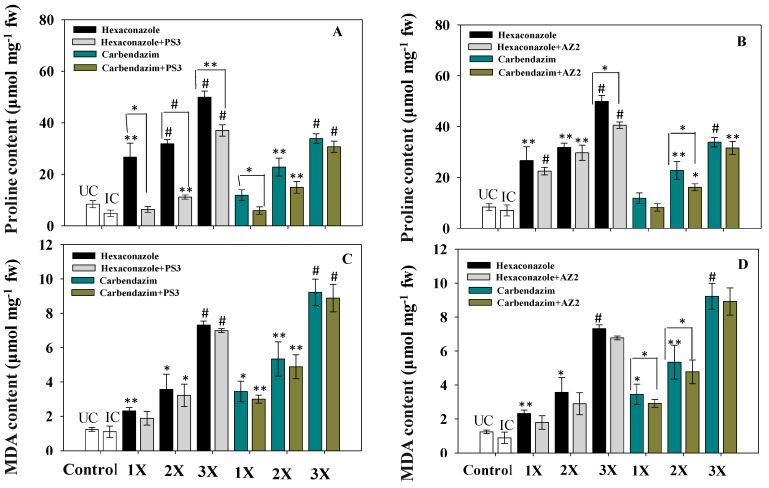
Bio-inoculation impact of *Pseudomonas* sp. PS3 (**A**) and *Pseudomonas* sp. AZ2 (**B**) on proline content accumulated in fresh foliage (fw) of *R. sativus* grown in soil treated with varying concentrations of carbendazim and hexaconazole. Inoculation impact of *Pseudomonas* sp. PS3 (**C**) and *Pseudomonas* sp. AZ2 (**D**) on malondialdehyde (MDA) content of radish foliage raised in soils treated with different concentrations of carbendazim and hexaconazole. Histograms represent the mean value of three independent replicates (*n* = 3), and error bars represent S.D. The asterisks *, ** and # denote statistical significance at *p* < 0.05, *p* < 0.005 and *p* < 0.001, respectively computed by Student’s *t*-test. UC = Uninoculated control, IC = Inoculated control. 1X, 2X and 3X denote the normal, two times and three times greater concentrations of fungicide.

Proline is often considered a stress biomarker, which is generated by plants under harsh conditions; it serves to protect cell membranes from the damaging impacts of the stressor by acting as a scavenger molecule [74]. Additionally, it can function as a protein-compatible hydrotrope and as a hydroxyl radical scavenger [75]. The enhancement of free cellular proteins under different biotic and abiotic stresses provides a number of defensive roles in most plant species [76]. A substantial accumulation of proline in fresh foliage of *R. sativus* grown under fungicide stress was recorded (Figure 9); proline concentration increased with increased fungicide dose. Maximum accumulation of 34 and 50 mg g^−1^ fresh weight was noted in foliage at the 3X the dose of CBZM and HEXA, respectively, which are 82% (*p* < 0.001) and 73% (*p* < 0.001) increases of proline as compared to proline accumulation in untreated (9 mg g^−1^ fresh weight) foliage (Figure 9). Increased proline accumulation in *R. sativus* grown under stressed conditions has been reported elsewhere [77]. In the presence of fungicide-tolerant PGPR, proline level was substantially reduced. *Pseudomonas* sp. PS3 reduced proline level to 34% (*p* < 0.005) when applied with 2X CBZM relative to uninoculated and treated with 2X CBZM (Figure 9A). *Pseudomonas* sp. AZ2 significantly (*p* ≤ 0.05) decreased proline content in plants dosed with 2X CBZM over the uninoculated control (Figure 9B). The reduction in proline content in *R. sativus* plants amended with PGPR isolates and supplemented with variable doses of fungicides is likely due to the detoxification potential of the bacterial isolates. Reduced proline levels in PGPR-inoculated plants cultivated in fungicide-stressed environments have been reported by various workers [21,78,79].

### 3.8. Lipid Peroxidation (MDA Content)

In plants, MDA is produced as a breakdown product of polyunsaturated fatty acid (PUFA) components of the lipid membrane [80]. Since MDA may serve as a stress biomarker of oxidative damage in plants, the level of MDA in fresh foliage of radish plants was evaluated. Lipid peroxidation in fungicide-treated and PGPR-inoculated radish plants increased slightly with an increase in fungicide concentration. Both isolates marginally reduced lipid peroxidation associated with the three concentrations (1X, 2X, and 3X) of each fungicide (Figure 9C,D). At the 3X dose of HEXA, maximum induction (9.8 µmol g^−1^ fresh weight) in MDA content was observed in radish foliage. An increase in lipid peroxidation level in radish foliage cultivated in greenhouse conditions and treated with copper was recently reported [81]. Similarly, Cura et al. [82] reported enhancement in MDA content of two common bean cultivars grown under stressed conditions.

Inoculation of PGPR isolates significantly reduced MDA levels by relieving fungicidal effects. *Pseudomonas* sp. AZ2 reduced MDA levels by 12% and 10% when applied to plants treated with 1X concentrations each of carbendazim and hexaconazole, respectively (Figure 9D). Similarly, a reduction of 4% (*p* < 0.001) and 5% (*p* < 0.001) in MDA content was recorded in plants inoculated with *Pseudomonas* sp. PS3 and treated with 10 mg kg^−1^ soil each of carbendazim and hexaconazole, respectively, over uninoculated but treated with identical doses of fungicide (Figure 9C). Reduction in MDA content following inoculation of stress-tolerant PGPR isolates to different vegetable crops such as *Lycopersicum esculentum* [83], *Abelmoschus esculentus* [84], *Cucumis sativus* [85], *Pisum sativum* [46], and *Raphanus sativus* [23] grown in the presence of various environmental pollutants has been reported.

### 3.9. Antioxidant Enzymes

**Figure 10:**
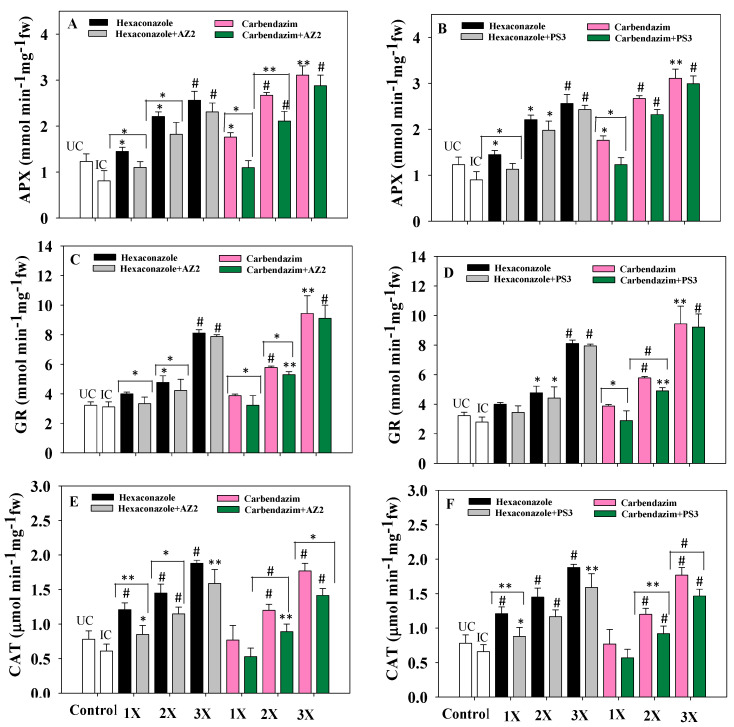
Effects of inoculation of fungicide-tolerant *Pseudomonas* sp. AZ2 on ascorbate peroxidase (APX) (**A**), glutathione reductase (GR) (**C**) and catalase (CAT) (**E**) activities in fresh foliage (fw) of radish plants grown in soil treated with carbendazim and hexaconazole. Panels (**B**,**D**,**F**) depict the impact of *Pseudomonas* sp. PS3 on APX, GR and CAT activities, respectively, in the presence of fungicides. Each value is a mean of three replicates (*n* = 3). Histograms represent the mean value of three independent replicates (*n* = 3) and error bars represent S.D. The asterisks *, ** and # denote statistical significance at *p* < 0.05, *p* < 0.005 and *p* < 0.001, respectively computed by Student’s *t*-test. UC = Uninoculated control, IC = Inoculated control. 1X, 2X and 3X denote the normal, two times and three times greater concentrations of fungicide.

Antioxidant enzymes such as ascorbate peroxidase (APX), superoxide dismutase (SOD), catalase (CAT), and glutathione reductase (GR) are generated by many cellular organelles like mitochondria and chloroplast and known for their indispensable roles in the antioxidant defense of biological systems [86]. ROS accumulation results in the activation of SOD, which leads to the synthesis of H_2_O_2_, a toxic signal molecule for oxidative stress [87]. Accumulation of H_2_O_2_ increases peroxidase and catalase activities in order to decrease H_2_O_2_ concentration by converting it to O_2_ and H_2_O [88]. Levels of antioxidant enzymes in radish leaf tissue increased as fungicide concentration increased from lower (1X) to higher (3X) concentrations. Among both fungicides, generally, hexaconazole imparted a greater toxic effect. Higher concentrations (3X) of fungicide were associated with maximum accumulation of antioxidant enzymes. Application of fungicide-tolerant PGPR reduced the toxicity and decreased levels of antioxidant enzymes. At the 3X concentration of HEXA, *Pseudomonas* sp. AZ2 reduced levels of APX (Figure 10A), GR (Figure 10C) and CAT (Figure 10E) by 10% (*p* < 0.001), 7% (*p* < 0.001) and 9.6% (*p* < 0.005), respectively, over uninoculated plants but treated with a similar concentration of fungicide. Similarly, activities of APX (Figure 10B), GR (Figure 10D) and CAT (Figure 10F) declined by 6.5% (*p* < 0.001), 4% (*p* < 0.001) and 18% (*p* < 0.001), respectively, when isolate PS3 was applied to radish grown in soil treated with 3X CBZM, compared to uninoculated plants treated with the same dose of fungicide. The reduced expression of antioxidative defense enzymes in fungicide-tolerant PGPR-inoculated radish plants may be related to mitigating fungicide toxicity and subsequently reducing oxidative damage. Decreases in antioxidant status of plants cultivated in pesticide-supplemented soil following inoculation of the pesticide-tolerant bacterium *Burkholderia cepacia* PSBB1 has been reported [41].

**Figure 11:**
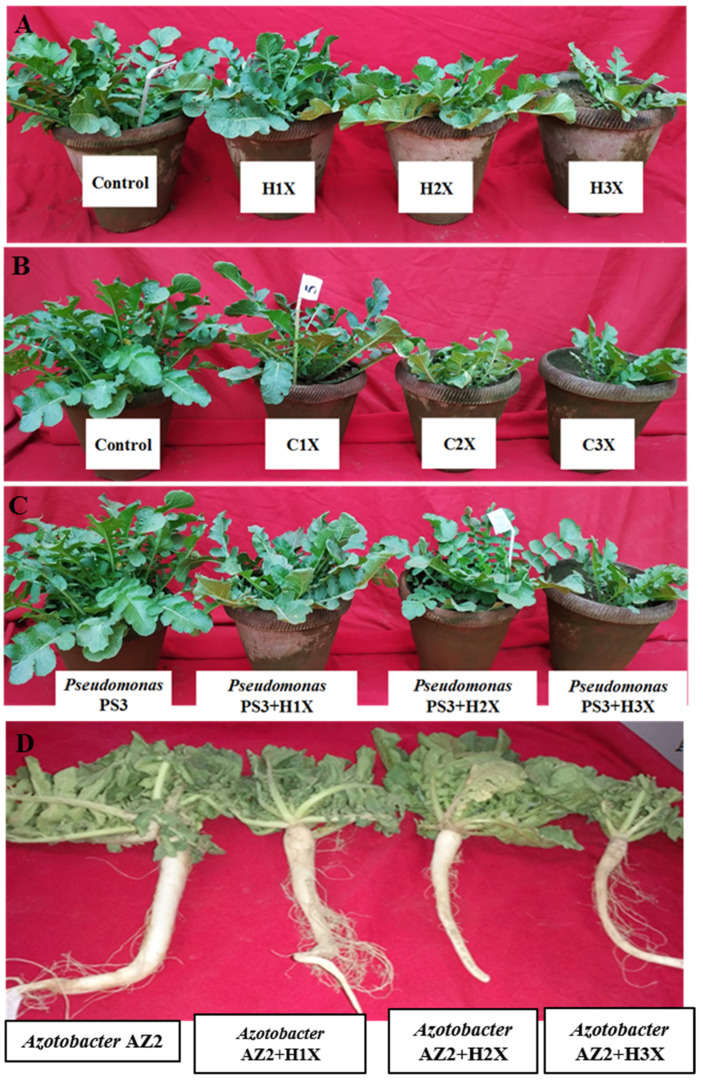
Effect of different concentrations of hexaconazole (**A**) and carbendazim (**B**) on growth of *R. sativus* grown in greenhouse conditions. Bioinoculation impact of *Pseudomonas* sp. PS3 on *R. sativus* grown in soil treated with 1X, 2X and 3X concentrations of hexaconazole (**C**). The panel (**D**) represent the roots system of *R. sativus* grown in soil treated with hexaconazole and inoculant AZ2. Here, H and C represent hexaconazole and carbendazim, respectively. The 1X, 2X and 3X represent the normal, two times greater and three times greater than normal application rate of fungicide.

In this study, tolerance of PGPR isolates to fungicides was variable (Figure 11), which could be due to variations in the genetic makeup of the test bacterial strains [89]. To overcome fungicidal stress and to safeguard the host plant, three mechanisms may be employed by PGPR, i.e., degradation of fungicides by enzymes, accumulation within cells followed by complexation with cellular constituents, and genetic mutation [90]. This unique intrinsic feature, which enables PGPR to tolerate high dosages of certain fungicides, could be exploited for sustainable agriculture via: (i) simple adaptation to soil contaminated with high levels of fungicides; (ii) revival or restoration of soil fertility; and (iii) following successful establishment, fungicide-tolerant PGPR can supply various bioactive compounds and benefit edible plants cultivated in contaminated soils. Certain compounds other than regularly produced bioactive molecules, for example, HCN, can selectively kill phytopathogens, thus protecting plants from the deleterious impact of fungicides while simultaneously enhancing crop yield [91].

## 4. Conclusions

The toxic and inhibitory impacts of fungicides on persistence and bioactive molecule-producing ability of *Pseudomonas* sp. AZ2 and *Pseudomonas* sp. PS3 varied significantly. Both PGPR isolates synthesized plant growth regulators even at high doses of fungicides; however, the quantity of plant growth-regulating substances declined substantially with increasing fungicide rate. Therefore, efforts must be directed to identify strategies regarding how secretion of plant growth-promoting substances could be preserved among PGPR while growing under harsh environments for long periods. Moreover, both isolates substantially mitigated the toxic effect of fungicides when applied as a biological inoculant to the radish crop. The noticeable enhancement in PGPR-supported radish plants treated with fungicides is encouraging and could be due to: (i) the inherent toxicity-mitigating properties of bacterial isolates and (ii) continued secretion and supply of growth-regulating substances by PGPR isolates to the radish crop even in a stressed environment. Based on these intrinsic features, it is strongly be suggested that AZ2 and PS3 isolates could safely and inexpensively be used as biofertilizers for augmenting the production of radish plants even in soils contaminated with fungicides.

## Figures and Tables

**Table 1:** Morphological and biochemical characteristics of plant growth-promoting rhizobacteria.

Characteristic	Plant Growth-Promoting Rhizobacteria	
	Isolate AZ2	Isolate PS3
Colony morphology	Irregular margin, white and mucoid colony	Irregular margin, mucoid pale yellow colony
Gram reaction	Negative	Negative
Cell shape Pigmentation	Short rod Non- fluorescent	Short rod Green fluorescent
Citrate utilization	+	+
Indole production	+	ND
Methyl red test	+	+
Nitrate reduction	+	+
Oxidase activity	ND	+
Voges Proskauer test	+	ND
Dextrose utilization	ND	+
Lactose utilization	ND	+
Mannitol utilization	+	+
Sucrose utilization	+	+
Starch hydrolysis	+	+
Gelatin hydrolysis	+	+
Maximum tolerance dose exposed to carbendazim	2400 µg mL^−1^	2400 µg mL^−1^
Maximum tolerance dose exposed to hexaconazole	1600 µg mL^−1^	3200 µg mL^−1^

‘+’ indicates ‘positive reaction’ whereas, ‘ND‘ represents ‘not detected’ (negative reaction).

**Table 2:** Plant growth-promoting activities of bacterial isolates under fungicide-stress.

Bacterial Isolate	Treatment	Plant Growth-Promoting Activity					
		Dose Rates (µg mL^−1^)	IAA (µg mL^−1^)	Phosphate-Solubilization (µg mL^−1^)	Siderophore Production	NH_3_ Production	HCN
*Pseudomonas* sp. PS3	Control	0	61.3 ^a^ ± 3.5	36.0 ^a^ ± 2.3	++	+++	++
	Carbendazim	500	49.4 ^c^ ± 2.3	28.8 ^b^ ± 3.0	+	++	+
		1000	39.8 ^d^ ± 2.8	21.0 ^c^ ± 1.3	+	+	+
		1500	21.3 ^e^ ± 2.0	15.1 ^d^ ± 1.2	ND	+	+
	Hexaconazole	500	56.3 ^b^ ± 3.2	28.9 ^b^ ± 3.2	+	++	++
		1000	40.0 ^d^ ± 3.0	21.3 ^c^ ± 1.7	+	+	+
		1500	24.8 ^e^ ± 1.3	11.8 ^d^ ± 1.4	ND	+	+
**Mean**	-	-	41.7	20.5	-	-	-
*Pseudomonas* sp. AZ2	Control	0	39.5 ^a^ ± 0	26.2 ^a^ ± 2.3	+++	+++	++
	Carbendazim	500	33.9 ^b^ ± 1.3	25.8 ^b^ ± 3.0	++	++	+
		1000	26.4 ^cd^ ± 1.7	20.0 ^c^ ± 1.3	+	+	+
		1500	18.9 ^e^ ± 2.3	14.4 ^d^ ± 1.2	+	+	+
	Hexaconazole	500	29.5 ^c^ ± 3.3	23.1 ^b^ ± 3.2	++	++	++
		1000	23.0 ^d^ ± 1.0	19.3 ^c^ ± 1.7	+	+	+
		1500	16.9 ^e^ ± 2.0	14.6 ^d^ ± 1.4	+	+	+
**Mean**	-	-	26.8	20.48	-	-	-
LSD (*p* ≤ 0.05)	-	-	92.46	45.4	-	-	-
F value	-	-	8.41	13.28	-	-	-

Each value is a mean of three replicates (*n* = 3). Mean values (mean ± S.D) are significant at *p* ≤ 0.05. Means followed by the same letter are significantly different according to Duncan’s multiple range test (DMRT). IAA: Indole-3-acetic acid; P: Phosphate; HCN: Hydrogen cyanide; +: low production, ++: moderate production, +++: high production, ‘ND’ ‘not detected’ and ‘LSD’ represents ‘Least significant difference’. The superscript letters a, b, c, d and e represents the significant difference in mean values at *p* ≤ 0.05.

## Supplementary Materials

The following are available online at https://www.mdpi.com/1660-4601/17/19/7251/s1, Table S1: Physico-chemical characteristics of fungicides used in the present study, Table S2: Physicochemical properties of test soil, Table S3: Inoculation impact of fungicide tolerant *Pseudomonas* sp. AZ2 on biological parameters of *R. sativus* grown in sandy clay loam soil treated with/without carbendazim and hexaconazole under pot house conditions, Table S4: Inoculation impact of fungicide tolerant *Pseudomonas* sp. PS3 on biological parameters of *R. sativus* grown in sandy clay loam soil treated with/without carbendazim and hexaconazole under pot house conditions.
