# Supplementary material for: Fungicide-Tolerant Plant Growth-Promoting Rhizobacteria Mitigate Physiological Disruption of White Radish Caused by Fungicides Used in the Field Cultivation
Source: Int J Environ Res Public Health. 2020 Oct 4;17(19):7251. doi: 10.3390/ijerph17197251 (PMC7579310; doi:10.3390/ijerph17197251)
Supplement: Supplementary file 1 [file ijerph-17-07251-s001.pdf]

Supplementary Materials

Fungicide-Tolerant Plant Growth Promoting Rhizobacteria Mitigate Physiological Disruption of White Radish Caused by Fungicides used in the Field Cultivation

Sadaf Khan [1], Mohammad Shahid [1,*], Mohammad Saghir Khan [1], Asad Syed [2], Ali H. Bahkali [2] and Abdallah M. Elgorban [2], John Pichtel [3]

[1] Department of Agricultural Microbiology, Faculty of Agricultural Sciences, Aligarh Muslim University, Aligarh-202002; Uttar Pradesh, India; sadaf4393@gmail.com (S.K.); khanms17@rediffmail.com (M.S.K.)

[2] Department of Botany and Microbiology, College of Science, King Saud University, P.O. 2455, Riyadh 11451, Saudi Arabia; asadsayyed@gmail.com (A.S.); abahkali@ksu.edu.sa (A.H.B.); aelgorban@ksu.edu.sa (A.M.E.)

[3] Natural Resources and Environmental Management, Ball State University, Muncie, IN, 47306, USA; jpichtel@bsu.edu (J.P)

* Correspondence: shahidfaiz5@gmail.com, Phone: +91 08090939511

Table S1. Physico-chemical characteristics of fungicides used in the present study.

Characteristics	Carbendazim	Hexaconazole
**Common name**	Carbendazim	Hexaconazole
**Chemical name**	methyl benzimidazol-2ylcarbamate	(RS)-2-(2,4-dichlorophenyl)-1-(1H-1,2,4triazol-1-yl) hexan-2-ol
**Chemical family**	Benzimidazole	Conazole
**Grade**	Commercial (50% w/w)	Commercial (5% SC
**Trade name**	Bendaco	Trigger, Bayer, Du pont
**Recommended dose**	1000 µg/kg	40 µg/kg
**Appearance**	White, crystalline solid	White milkish liquid
**Molecular weight**	191.187g/mol	314.21
**Empirical formula**	$C_9H_9N_3O_2$	$C_{14}H_{17}C_{l2}N_3O$
**Solubility**	Water/DMSO	Water, acetone, methanol. Etc
**Source**	Agrochemicals, New Delhi, India	Parijat Agrochemicals, New Delhi, India

Table S2. Physicochemical properties of test soil.

Soil characteristics	Unit	Value
Organic carbon	g kg^{-1}	6.2
Kjeldahl nitrogen	g kg^{-1}	0.75
Olsen phosphorus	mg kg^{-1}	16
Water holding capacity	mL g^{-1}	0.44
Cation exchange capacity	cmol kg^{-1}	11.7
Anion exchange capacity	cmol kg^{-1}	5.7

Supplementary Methods

2.4. Morphological characterization and Biochemical Reactions

The biochemical tests employed for presumptive identification of PGPR strain PS3 and AZ2 included: citrate utilization, indole production, methyl red, nitrate reduction, oxidase test, Voges Proskauer, sugar fermentation, starch hydrolysis and gelatine liquefaction (Holt et al. 1994).

2.4.1 Gram reaction

In order to categorise heterogeneously distributed bacterial populations in to Gram positive and Gram-negative groups, bacterial cultures recovered from various rhizosphere sites were grown overnight and were subjected to Gram reaction. The bacterial populations

appearing purple and red under simple microscope were grouped as Gram positive and Gram-negative bacteria, respectively. The colony characteristics of bacterial cells such as size, shape, margin and pigmentation etc. were also recorded.

2.4.2 Citrate utilization test

Autoclaved Simmons's citrate agar plates were spot inoculated with test isolates and incubated at 28±2 ^{0}C for 24-48 h. Change in colour from green to blue was observed.

2.4.3 Indole reaction

Using autoclaved nutrient broth each test isolate was incubated at 28±2 $^{\circ}$C for 24-48 h. After incubation 2-3 drops of Kovac's reagent was added to broth and observed for the formation of red ring.

2.4.4 Methyl red test

Autoclaved MR-VP broth inoculated with each isolate was incubated at 28±2 ^{0}C for 24-48 h. Methyl red solution was added as indicator. The development of colour from red to nil was considered as positive and negative reactions.

2.4.5 Nitrate reduction test

Autoclaved trypticase nitrate broth tubes inoculated with test isolates were incubated at 28±2^{0}C for 24-48 h. Five drops of solution A and few drops of solution B were added and examined for formation of red colour.

2.4.6 Oxidase test

Oxidase disc was moistened with distilled water. Cultures were spread on plates containing oxidase disc. The colour of the disc changed from deep blue to deep purple indicating a positive test, while the colourless spot indicated the negative test.

2.4.7 Voges-Proskauer test

Autoclaved MR-VP broth was inoculated with test organism and incubated at 28±2 ^{0}C for 24-48 h. After incubation, Barrit's reagent was added and observed for red colour formation.

2.4.8 Sugar fermentation test

Autoclaved fermentation broth (Phenol red broth) supplemented with 7 g/L of dextrose, lactose, mannitol and sucrose was inoculated with test isolates and incubated at 28±2^{0}C for 24-48 h. Production of acid or acid with gas was observed.

2.4.9 Starch hydrolysis test

Autoclaved starch agar plates were spot inculcated with test isolates and incubated at 28±2^{0}C for 24-48 h. After incubation, plates were flooded with iodine solution. Clear zone of hydrolysis around the growth was observed.

2.4.10 Gelatin hydrolysis

Tubes containing autoclaved nutrient broth, amended with 12% gelatin tubes were inoculated with test isolates and incubated at 28±2^{0}C for 48 h. After incubation, tubes were placed at 4 ^{0}C for 30 min. On refrigeration, liquefied tubes indicated positive test.

2.5 Identification of rhizobacterial strains using 16SrRNA gene sequencing

The total genomic DNA was extracted by the method as previously described by Yadav et al. (2011). The forward primer pA (5'AGA GTT TGA TCC TGG CTC AG3') and reverse primer pH (5'AAG GAG GTG ATC CAG CCG CA3') (Solanki et al. 2012) were used to amplify the 16S rRNA gene from genomic DNA. The total volume of reaction mixture was 100 µl containing 50–80 ng of template DNA, 10X reaction buffer, 2.5mM dNTPs, 20 pM of each primer and one unit Taq DNA polymerase (Bangalore Genei, India), and

reactions were performed on G-storm thermocycler (G-STORM, UK). The amplification conditions were as follows: initial denaturation at 94°C for 5 min, followed by 35 cycles of denaturation at 94°C for 40 s, annealing at 52 °C for 45 s, and elongation at 72 °C for 1min 30s. At the end of 35 cycles, the final extension step was at 72 °C for 8 min. The amplified product was resolved by electrophoresis in 1.2% agarose gel in 1X TAE buffer. Gels were stained with ethidium bromide (10 mg ml^{-1}) and visualized on gel documentation system (BIO-RAD, USA). Strong and clear bands were scored for similarity and clustering analysis using the software, NTSYS-2.02e package (Numerical taxonomy analysis program package, Exeter software, USA). The purified16S rRNA PCR products were send to Macrogen, Seol, south Korea for 16S rRNA sequencing. The 16S rRNA amplicons were sequenced from both ends and consensus sequence was generated. The partial 16S rRNA gene sequences were compared with those available in the databases (http://www.ncbi.nlm.nih.gov/BLAST/) and identification to the species level was determined on the basis of sequence similarity of >97% with the closest relative in the GenBank. The phylogenetic tree was constructed on the aligned datasets using the neighbour-joining method implemented in the program MEGA 4.0.2 (Tamura et al. 2007). Bootstrap analysis was performed on 1,000 random samples taken from the multiple alignments.

2.5.1. Bioassay of Indole acetic acid (IAA)

Indole-3-acetic acid (IAA) produced by fungicide tolerant bacterial strain was quantitatively assayed by the modified method of Brick et al. (1991). Here, bacterial isolates were grown in Luria Bertani (LB) broth (gl^{-1}: tryptone 10; yeast extract 5; NaCl 10 and pH 7.5). A- 100 ml LB broth containing fixed concentration of tryptophan (100 mg ml^{-1}) was treated with 1X, 2X and 3X concentrations each of CBZM and HEXA. The fungicide containing LB was then inoculated with 100 μL culture (10^8 cells ml^{-1}) of bacterial strains and

incubated at 28±2 °C for four days with shaking at 120 r/min. Following complete incubation, culture (5 ml) was centrifuged (8000 r/min) for 10 min. and two ml supernatant was added with 100 µL orthophosphoric acid and four ml Salkowsky reagent (2% 0.5 M $FeCl_3$ prepared in 35% per-chloric acid) and incubated for one hour at 28±2°C in dark for colour development. The absorbance of pink colour developed during reaction was measured at 530 nm. The quantity of indole acetic acid was calibrated using pure IAA as a standard.

2.6.1 Morphological Distortion in PGPR induced by fungicides under SEM

To assess the effect of fungicides CBZM and HEXA on surface morphology of both test PGPR strains (AZ2 and PS3), scanning electron microscopy was done. Fungicide induced distortion in AZ2 and PS3 strains was observed under SEM (Shahid et al., 2019) by growing the bacterial strains in NB medium treated with 1500 μgmL^{-1} each of HEXA and CBZM at 28±2°C for 24 h. The cultures grown in fungicide free environment were served as control. After incubation, cultures were centrifuged at 12,000 rpm for 10 min. and pellet was suspended in 1× PBS and the cell pellet were washed again three times with 1× PBS and pre-fixed with 2.5% glutaraldehyde for overnight at 4°C. The cells were recovered by centrifugation at 10000 rpm for 5 min. and pellet was again washed with same buffer. After three successive washing, the fixed specimens were dehydrated in a graded series (30, 50, 70, 90 and 100%) of ethanol for 5 min. each. After this, cell pellets were centrifuged and re-suspended in PBS. Five µL of bacterial suspension was smeared on coverslip and dried. The specimens were mounted and analysed under the SEM to see the changes in bacterial structures, if any, and images were recorded.

2.7.1 Biological attributes of greengram plants: Germination Efficiency and Seedling vigor index

Healthy greengram seeds were surface sterilized using 3% (w/v) sodium hypochlorite (NaOCl) for three min. followed by three successive washing with DW. Five days after sowing (DAS), radicle emergence of one millimetre was considered as germinated seeds and germination percentage was calculated as:

$$\text{Germination \%} = \frac{\text{Number of seeds germinated}}{\text{Total number of seeds}} \times 100$$

The percentage of germination was used further to calculate the seedling vigor index (Abdul-Baki and Anderson, 1973)

$$\text{Seedling vigor Index (SVI)} = [RL + SL] \times \text{\% Seed Germination}$$

2.7.3 Effect of PGPR inoculants on photosynthetic pigments

Photosynthetic pigments (total chlorophyll and carotenoid content) in fresh radish foliage of was measured according to the method of Arnon (1949). The pigments were extracted from fresh leaves by macerating in 80% acetone. Absorption of chlorophyll and carotenoid content in the extract was determined using UV visible spectrophotometer (UV-2450, Shimadzu, Tokyo, Japan). The total photosynthetic pigments (Chl a, Chl b and total chlorophyll) was calculated as:

$$\text{mg chl. a/g tissue} = 12.7\ (A_{663}) - 2.69\ (A_{645}) \times V/1000 \times W$$

$$\text{mg chl. b/g tissue} = 22.9\ (A_{645}) - 4.68\ (A_{663}) \times V/1000 \times W$$

$$\text{mg total chl./g tissue} = 20.2\ (A_{645}) + 8.02\ (A_{663}) \times V/1000 \times W$$

Carotenoid content was determined by the formula as suggested by Kirk and Allen (1965):

$$\text{Carotenoids (mg/g tissue)} = (A_{480}) + 0.114\ (A_{663}) - 0.638\ (A_{645})$$

Where, A_λ = absorbance at specific wavelength λ (nm); V= final volume of chlorophyll extracted in 80% acetone, and W= fresh weight of tissue extract.

2.8.1 Free Proline Content in radish foliage

Briefly, one-gram of fresh plant organ was homogenized with 5 mL of 3% (w/v) aqueous sulfosalicylic acid ($C_7H_6O_6S$). The resulting homogenate was filtered through Whatman No.2 filter paper. The resulting cell extract was then centrifuged at 8000 rpm for 20 min, to remove cell debris. The cell filtrate (2 mL) with free proline was treated with 2 mL of acid ninhydrin and glacial acetic acid (2 mL) at 80 °C for one h. The mixture was heated in boiling water bath for one hour. The reaction was terminated by placing the tubes in ice bath. A- 4 mL of toluene was added to the reaction mixture and stirred well for 20 to 30 seconds. Coloured complex was extracted in toluene and the toluene layer was separated. The red colour intensity was measured at 520 nm. A series of standard with pure proline was run in a similar way by dissolving proline in 3% $C_7H_6O_6S$ and a standard curve was prepared. Amount of free proline in the test sample was determined from the standard curve. Proline content on fresh weight basis (µ moles per gram of fresh weight of tissue) was expressed as:

$$\text{Proline content } = \frac{\mu g\ proline/\ ml\ \times ml\ of\ toluene}{115.5} \times \frac{5}{g\ of\ sample}$$

Where, 115.5 is the molecular weight of proline.

2.8.3 Extraction and Determination of Antioxidant Enzymes

For antioxidant enzyme activity, foliage was crushed in 4 mL of enzyme extraction buffer [(50 mM phosphate buffer (pH=7.8)] containing 1mM EDTA and 2% (w/v) polyvinylpyrrolidone (PVP). For GPX (E.C. 1.11.1.7), foliage tissues (100 mg) were homogenised in tris buffer and the homogenate was centrifuged at 12,000 rpm for 20 min. at 4 ºC. Increase in absorbance at 470 nm due to formation of ttra guaiacol (ε = 26.6 mM^{-1} cm^{-1}) is expressed as µ mol mg $protein^{-1}$ min^{-1}. The reaction mixture (3 mL) consisted of 100 mM phosphate buffer (pH=7.0), 0.1 mM EDTA and 20 mM H_2O_2. The reaction was initiated by adding 100 µL of enzyme extract. All enzyme assays were performed three times with three replicates of each assay.

2.8.3.1 ***Catalase (CAT) activity***

CAT activity was measured according to the method of Beer and Sizer (1952), with minor modifications. The reaction mixture (3 mL) consisted of 100 mM phosphate buffer (pH=7.0), 0.1 mM EDTA and 20 mM H_2O_2. The reaction was initiated by adding 100 µL of enzyme extract. The decrease in H_2O_2 was monitored at 240 nm at 1 min interval up to five min and CAT activity was quantified by using the molar extinction coefficient of H_2O_2 (36 $mol^{-1}cm^{-1}$).

2.8.3.2 ***Glutathione reductase (GR)***

For GR activity, 0.2 mL of enzyme extract was added to 2.8 mL reaction mix. (H_2O, buffer, 30 mM GSSG, 0.8 mM NADPH and 1 % BSA) and the absorbance was measured at 340 nm for 3 min at an interval of 30 sec. (Ahmed et al., 2018). The enzyme activity was expressed in µmol ascorbate oxidised min^{-1} g^{-1} dw.

2.8.3.4 ***Ascorbate peroxidase (APX)***

The APO activity was determined following the method as described by Nakano and Asada (1981).

Table S3. Inoculation impact of fungicide tolerant *Pseudomonas* sp. AZ2 on biological parameters of *R. sativus* grown in sandy clay loam soil treated with/without carbendazim and hexaconazole under pot house conditions.

Treatments		Dose rate(mg/kg)	Plant length (cm)		Plant fresh weight (g)			Plant dry weight (g)	
			**Root**	**Shoot**	**Root**	**Shoot**	**Whole**	**Root**	**Shoot**
Uninoculated			22.3^{b}	26.7^{b}	34^{c}	64.3^{b}	98.3^{b}	5.1^{b}	10.4^{b}
	**Control**	0							
	CBZM1X	2	17.3^{e}	21^{c}	29^{d}	49.6^{c}	78.6^{c}	3^{d}	7.3^{c}
	CBZM2X	5	13.6^{f}	17^{d}	24^{e}	43^{d}	67^{d}	2.2^{e}	6.4^{d}
	CBZM3X	10	9.3^{h}	11^{f}	22^{f}	30.7^{f}	52.7^{e}	1.2^{g}	3.2^{f}
	HEXA1X	2	19^{d}	23.6^{c}	24^{e}	43^{d}	67^{d}	4^{c}	5.2^{e}
	HEXA2X	5	16^{e}	18.6^{d}	21^{f}	35.6^{e}	56.6^{e}	3.2^{d}	4^{f}
	HEXA3X	10	11.6^{g}	14^{e}	14^{g}	24.4^{g}	38.4^{f}	1.6^{f}	2.1^{g}
**Inoculated**	*Pseudomonas* sp. AZ2	0	27.7^{a}	30.3^{a}	50^{a}	71.6^{a}	121.6^{a}	7.0^{a}	12^{a}
	AZ2+CBZM1X	2	18.3^{d}	22.6^{c}	36^{c}	55^{b}	91^{b}	3.8^{c}	8.5^{c}
	AZ2+CBZM2X	5	14.^{6}	18^{d}	27^{e}	47.6^{d}	74.6^{c}	2.7^{d}	6.8^{d}
	AZ2+CBZM3X	10	10^{h}	11.6^{f}	25^{e}	35.3^{e}	60.3^{e}	1.5^{f}	3.4^{f}
	AZ2+HEXA1X	2	21.8^{c}	25.5^{b}	43^{b}	59^{b}	102^{b}	5.1^{b}	5.5^{e}
	AZ2+HEXA2X	5	18.1^{d}	19.1^{d}	38^{c}	48.6^{c}	86.6^{c}	3.5^{c}	4.4^{e}
	AZ2+HEXA3X	10	14^{f}	14.3^{e}	32^{d}	36.6^{e}	68.6^{d}	1.8^{e}	2.4^{g}
**Mean value**		-	16.4	19.1	29.9	46.0	76.0	3.1	3.3
**LSD**		-	1.31	4.21	17.2	9.1	3.8	10.2	14.9
**F value**		-							
**Uninoculated (df = 1)**			36.2	76.3	57.9	11.2	43.2	63.2	0.92
**Inoculated (df = 1)**		-	56.2	66.1	85.2	0.67	45.3	3.21	7.32
**Uninoculated × inoculated (df = 3)**		-							
			2.43	21.2	45.2	8.21	33.1	42.1	1.56

Each value is a mean (mean ± S.D) of three independent replicate (n=3) where each replicate constituted three plants/pot. Mean values followed by different letters are significantly different within a row or column at p≤0.05. according to DMRT test. In this and subsequent tables, CBZM and HEXA represents the carbendazim and hexaconazole, respectively.

Table S4. Inoculation impact of fungicide tolerant *Pseudomonas* sp. PS3 on biological parameters of *R. sativus* grown in sandy clay loam soil treated with/without carbendazim and hexaconazole under pot house conditions.

Treatments		Dose rate(mg/kg)	Plant length (cm)		Plant fresh weight (g)			Plant dry weight (g)	
			Root	Shoot	Root	Shoot	Whole	Root	Shoot
**Un-inoculated**									
	**Control**	0	22.3^{b}	26.7^{b}	34^{b}	64.3^{b}	98.3^{b}	5.1^{a}	10.4^{b}
	CBZM 1X	2	17.3^{d}	21d^{e}	29^{c}	49.6^{c}	78.6^{c}	3^{d}	7.3^{c}
	CBZM 2X	5	13.6^{f}	17^{f}	24^{d}	43^{d}	67^{d}	2.2^{e}	6.4^{d}
	CBZM 3X	10	9.3^{h}	11^{h}	22^{e}	30.7^{f}	52.7^{f}	1.2^{f}	3.2^{g}
	HEXA 1X	2	19^{c}	23.6^{c}	24^{d}	43^{d}	67^{d}	4^{c}	5.2^{e}
	HEXA 2X	5	16^{e}	18.6^{e}	21^{e}	35.6^{e}	56.6^{f}	3.2^{d}	4^{f}
	HEXA 3X	10	11.6^{g}	14^{g}	14^{f}	24.4^{g}	38.4^{h}	1.6^{f}	2.1^{h}
**Inoculated**	*Pseudomonas* sp. PS3	0	29.3^{a}	32^{a}	36^{a}	69.7^{a}	105^{a}	6.4^{a}	11.2^{a}
	PS3+CBZM 1X	2	23^{b}	26.3^{b}	29^{c}	52^{c}	81^{c}	3.4^{d}	8.2^{c}
	PS3+CBZM 2X	5	19^{c}	20.3^{d}	24^{d}	45.6^{d}	69.6^{d}	2.5^{e}	7.1^{c}
	PS3+CBZM 3X	10	14.6^{f}	15.3^{g}	19^{f}	32.3^{e}	51.3^{f}	3.7^{c}	3.5^{g}
	PS3+HEXA 1X	2	22.3^{b}	27^{b}	28^{c}	45.0^{d}	73.0^{d}	4.9^{b}	7.2^{c}
	PS3+HEXA 2X	5	18.6^{c}	20^{d}	22^{e}	42.6^{d}	64.6^{e}	3.5^{d}	6.3^{d}
	PS3+HEXA 3X	10	14^{f}	15^{g}	15^{f}	28.3^{f}	43.3^{g}	1.9^{e}	2.4^{h}
**Mean value**		-	17.8	20.7	24.3	43.3	67.6	3.3	5.9
LSD		-	87	2.32	22.1	2.3	2.7	7.1	3.74
F value									
Un inoculated(df=1)		-	45.6	134	228	27.8	56.8	119.3	0.84
inoculated(df=1)		-	34.1	67.5	23.4	0.76	77.3	46.8	12.5
Un inoculated × inoculated (df=3)		-							
			76.3	28.1	18.2	12.5	89.4	71.8	0.56

Each value is a mean (mean ± S.D) of three independent replicate (n=3) where each replicate constituted three plants/pot. Mean values followed by different letters are significantly different within a row or column at p≤0.05. according to DMRT test. In this and subsequent tables, CBZM and HEXA represents the carbendazim and hexaconazole, respectively.

References

Abdul-Baki, A.A. and Anderson, J.D. Vigor determination in soybean seed by multiple criteria Crop Sci. 1973, 13, 630-633.

Ahmad, I.; Akhtar, M.J.; Mehmood, S.; Akhter, K.; Tahir, M.; Saeed, M.F.; Hussain, M.B.; Hussain, S. Combined application of compost and *Bacillus* sp. CIK-512 ameliorated the lead toxicity in radish by regulating the homeostasis of antioxidants and lead. Ecotoxicol. Environ. Safety 2018, 148, 805-812.

Arnon, D.I.; Copper enzymes in isolated chloroplasts. Polyphenoloxidase in *Beta vulgaris*. Appl. Environ. Microbiol. 1949, 55, 1665–1669.

Beers, R.F. and Sizer, I.W. A spectrophotometric method for measuring the breakdown of hydrogen peroxide by catalase. J Biol chem, 1952, 195, 133-140.

Brick, J.M.; Bostock, R.M.; Silverstone, S.E. Rapid *in situ* assay for indoleacetic acid production by bacteria immobilized on a nitrocellulose membrane. Appl. Environ. Microbiol 1991, 57, 535-538

Holt GJ, Krieg NR, Sneath PHA. Gram negative aerobic/ microaerophilic rods and cocci. In: Bergey's manual of determinative bacteriology, 9th edn. Williams and Wilkins, Lippincott, Philadelphia 1994

Kirk, J. T. O.; Allen, R. L. Dependence of chloroplast pigments synthesis on protein synthetic Selenium dioxide were powdered separately and mixed together effects on actilione. Biochem. Biophys. Res. J. Canada. 1965, 27, 523-530.

Nakano, Y. and Asada, K., 1981. Hydrogen peroxide is scavenged by ascorbate-specific peroxidase in spinach chloroplasts. Plant and Cell Physiol. 1981, 22, 867-880.

Shahid, M.; Khan, M.S.; Kumar, M. Kitazin-pea interaction: understanding the fungicide induced nodule alteration, cytotoxicity, oxidative damage and toxicity alleviation by *Rhizobium leguminosarum*. RSC Adv 2019a, 9, 16929-16947.

Solanki MK, Kumar S, Pandey AK, Srivastava S, Singh RK, Kashyap PL, Srivastava AK, Arora DK. 2012. Diversity and antagonistic potential of Bacillus spp. associated to the rhizosphere of tomato for the management of Rhizoctonia solani. Biocontrol Sci Technol 22: 203-217

Tamura K, Dudley J, Nei M, Kumar S. MEGA4, molecular evolutionary genetics analysis (MEGA) software version 4.0.2. Mol Biol Evol 2007, 24:1596–1599

Yadav S, Kaushik R, Saxena AK, Arora DK. Diversity and phylogeny of plant growth promoting bacilli from moderately acidic soil. J Basic Microbiol 2011, 51:98-106.
